# A 20.5 cm Malignant Peripheral Nerve Sheath Tumor of the Anterior Mediastinum Showing Response to Neoadjuvant Therapy

**DOI:** 10.7759/cureus.31299

**Published:** 2022-11-09

**Authors:** Malika P Ganguli, Jophil Thomas, Mahlon R Kile, Sri Yadlapalli

**Affiliations:** 1 Internal Medicine, Ross University School of Medicine, Bridgetown, BRB; 2 Internal Medicine, St. Joseph Mercy Oakland Hospital, Pontiac, USA

**Keywords:** sarcoma, rare tumors, anterior mediastinum, neurofibromatosis 1 (nf1), malignant peripheral nerve sheath tumor (mpnst)

## Abstract

Malignant peripheral nerve sheath tumors (MPNST) are a rare form of sarcoma derived from Schwann cells. Major risk factors for development are neurofibromatosis 1 (NF1) and prior radiation exposure. Tumor location is highly variable. We present a case of an extremely large MPNST tumor in the anterior mediastinum in a 66-year-old male. To the best of our knowledge, the 20.5 cm tumor is the first of its kind in a patient without clinical signs of NF1 or prior radiation exposure. The localization of this tumor to the anterior mediastinum is rarer, as the most common tumors presenting in this area are thyroid neoplasms, thymomas, teratomas, and lymphomas. The patient’s tumor responded to doxorubicin-ifosfamide-mesna-based therapy. The tumor decreased from 20.5 cm to 9.0 cm on subsequent imaging. Thus, this is an interesting and valuable case to learn about the presentation and potential treatments of such a rare pathology.

## Introduction

Malignant peripheral nerve sheath tumors (MPNST) are rare. The tumor is diagnosed using any one of four major criteria: peripheral nerve origin, arising from a pre-existing tumor, history of Neurofibromatosis Type 1 (NF1), or pathology-proven Schwann cell origin [1-3]. NF1 is a known risk factor for MPNST, caused by a mutated neurofibromin protein [4-7]. NF1 is recognized by cafe-au-lait spots, Lisch nodules, optic gliomas, cutaneous tumors of peripheral nerve origin, and intellectual disability, amongst other signs. Radiation exposure is also a risk factor; however, only 10% of tumors arising from this etiology are MPNST [4]. Intrathoracic MPNST, especially anterior mediastinal cases, forms a diagnostic and treatment challenge due to its rarity and scarce literature. Thus, this is an important learning case to understand and treat patients with similar conditions. This article was previously presented as an abstract at the American College of Physicians' Michigan Chapter Meeting on October 15, 2022.

## Case presentation

**Figure 1:**
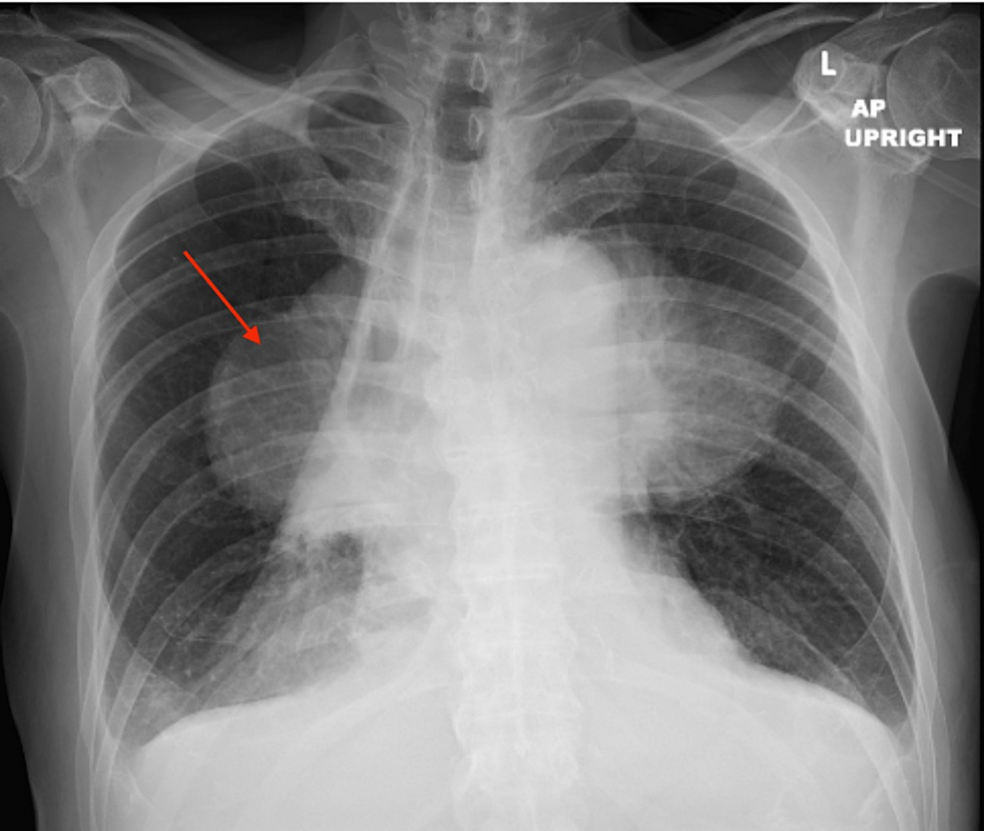
Initial chest X-ray A large ovoid opacity extending across bilateral midlungs and mediastinum is indicated by the red arrow, indicating a possible mass.

The patient is a 66-year-old Caucasian man with a 30-pack-a-year smoking history (he quit 14 years ago) and prior esophagectomy and gastric pull-through for high-grade premalignant esophageal lesions. The patient's cough, chest pressure, and shortness of breath worsened over time. He was initially treated on an outpatient basis for pneumonia. However, his symptoms continued for more than three months, which prompted further investigation. Initial plain film radiography demonstrated a large, ovoid-shaped opacity suggestive of a mass (Figure 1).]

**Figure 2:**
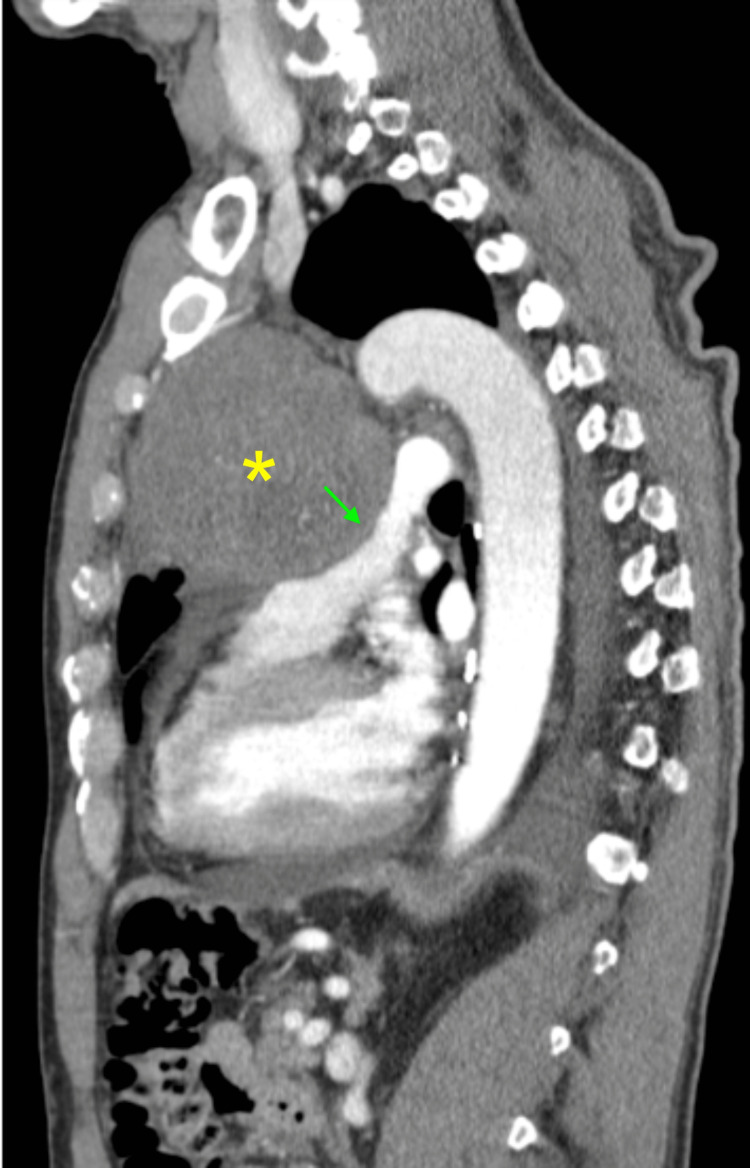
Chest CT, sagittal view The yellow asterisk (*) indicates a large anterior mediastinal mass. The green arrow indicates a mass effect on adjacent cardiopulmonary structures.

**Figure 3:**
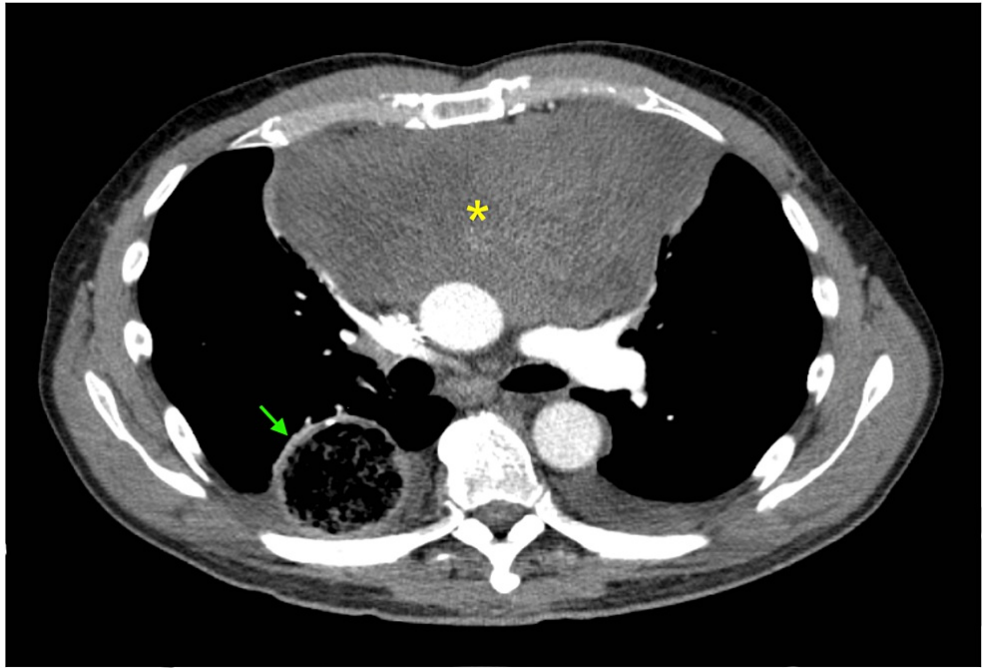
Chest CT, transverse view The yellow asterisk (*) indicates an anterior mediastinal mass. The green arrow indicates gastric pull-through remnants.

Subsequent computed tomography (CT) with the contrast of the chest showed a 9.0 x 20.5 x 9.2 cm mass in the anterior mediastinum containing calcifications, central necrosis, and mass effect on adjacent cardiopulmonary structures (Figures 2, 3).

**Figure 4:**
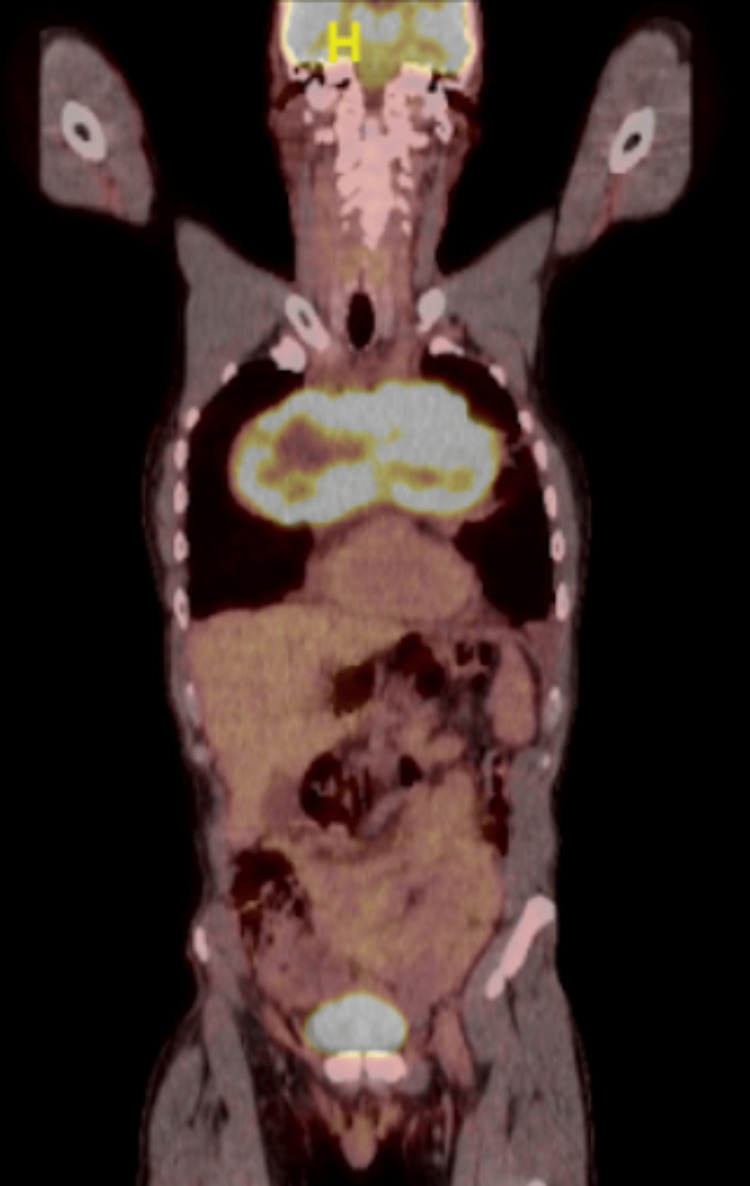
PET scan

A positron emission tomography (PET) scan showed fluorodeoxyglucose (FDG) uptake with a maximum standardized uptake value (SUV max) of 13 (Figure 4).

**Figure 5:**
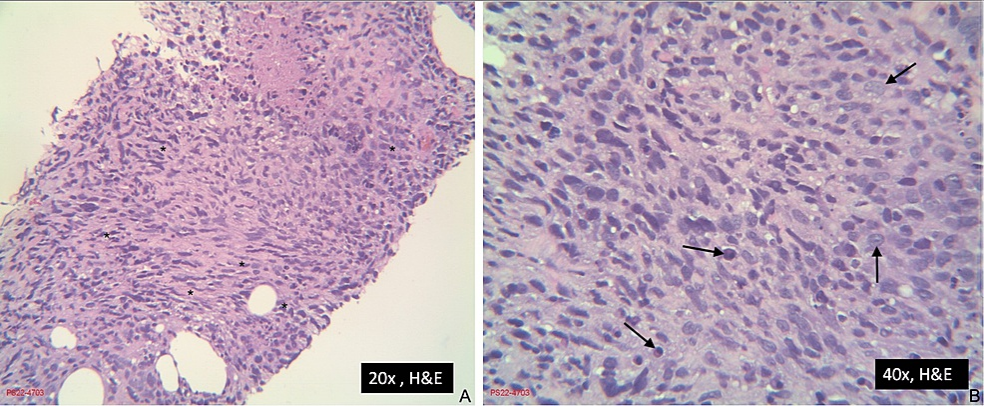
Malignant peripheral nerve sheath tumor biopsy Pane A (left) shows the tumor biopsy slide at 20x magnification using hematoxylin and eosin staining. The black asterisks (*) indicate areas of disorganized cell growth and palisading necrosis. Pane B (right) shows the tumor biopsy slide at 40x magnification using hematoxylin and eosin staining. The black arrows indicate cells in multiple stages of mitosis.

**Figure 6:**
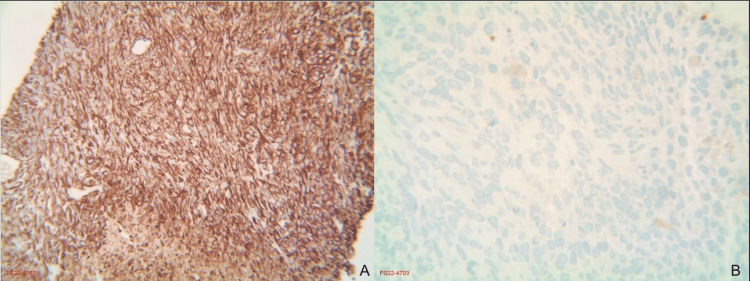
Immunohistochemical staining slides Pane A (left): positive for vimentin, at 40x magnification. Pane B (right): negative for H3K27me3, at 40x magnification.

Laboratory investigations demonstrated an elevated lactate dehydrogenase of 671 U/L (reference: 140-271 U/L), with normal alpha-fetoprotein (AFP) and beta-human chorionic gonadotropin (b-HCG). A core needle biopsy indicated high-grade sarcoma positive for vimentin with brisk mitotic activity, palisading necrosis, and immunohistochemical loss of H3K27me3 expression (Figures 5, 6, and Table 1). These findings confirm MPNST with grade 3 histology and staging as stage IIB (T2b, N0, M0, G2). This patient did not exhibit any clinical manifestations of NF1.

**Table 1:** Immunohistochemical markers tested in this case compared to those in literature. (++) denotes positive, (+) denotes weakly positive, (-) denotes negative, and (N/A) denotes not available. A-sma: alpha-type smooth muscle actin; BCL2: B-cell lymphoma 2; CD: cluster of differentiation; EMA: epithelial membrane antigen; OCT-4: cctamer-binding transcription factor; TTF1: thyroid transcription factor 1; WT-1: Wilms tumor protein 1

	This Patient	Shimoyama et al., 2009 [4]	Kalra et al., 2014 [5]	Koezuka et al., 2014 [6]	Rais et al., 2020 [7]
A-sma	N/A	-	-	-	-
BCL2	N/A	+	++	++	N/A
Calretinin	N/A	N/A	N/A	-	N/A
CD30	-	N/A	N/A	N/A	N/A
CD31	-	N/A	N/A	N/A	N/A
CD34	N/A	++	N/A	N/A	-
CD45	-	N/A	N/A	N/A	N/A
CD56	N/A	N/A	N/A	++	N/A
CD68	N/A	++	N/A	N/A	N/A
CD99	N/A	++	-	+	N/A
CD117	N/A	N/A	N/A	N/A	-
Chromogranin A	-	N/A	N/A	-	N/A
Cytokeratin 5	-	+	N/A	N/A	-
Cytokeratin 6	N/A	N/A	N/A	N/A	-
Cytokeratin 7	N/A	N/A	N/A	N/A	-
Cytokeratin 20	N/A	N/A	N/A	N/A	-
Desmin	-	-	-	-	N/A
EMA	-	-	-	N/A	-
H caldesmon	N/A	N/A	N/A	-	N/A
OCT-4	-	N/A	N/A	N/A	N/A
P40	-	N/A	N/A	N/A	N/A
S100	-	+	++	-	-
Synaptophysin	-	N/A	N/A	-	N/A
TTF1	N/A	N/A	N/A	N/A	-
Vimentin	++	++	++	++	++
WT-1	N/A	N/A	N/A	-	N/A

The patient received five cycles of neoadjuvant chemotherapy. The first round consisted of doxorubicin 75 mg/m2, ifosfamide 2500 mg/m2, mesna oral tablet 1000 mg/m2, and two infusion bags of mesna 500 mg/m2 per dose. The second, third, fourth, and fifth rounds of chemotherapy consisted of doxorubicin 75 mg/m2 with reduced dosages of ifosfamide at 2000 mg/m2, mesna oral tablet at 800 mg/m2, and two infusion bags of mesna at 400 mg/m2. The cumulative doxorubicin dose was documented at 375 mg/m2.

Follow-up imaging revealed that the tumor had shrunk to 9.0x6.1cm in size. Magnetic resonance imaging (MRI) of the chest confirmed no invasion into the intraluminal aorta or main pulmonary artery and demonstrated large surface area contact with the ascending aorta with no clear image to determine tumor invasion into the aortic wall. He did not receive radiation therapy or surgical intervention. Surgery is complicated due to the proximity of cardiopulmonary structures and the inability to evaluate a surgical fat plane. In addition to tumor size reduction, this patient reported marked clinical improvement. He experienced reduced pain, an improvement in hypoxia, the discontinuation of home oxygen use, and an increased tolerance for physical activity.

## Discussion

Malignant peripheral nerve sheath tumors are rare tumors, accounting for 4%-10% of soft tissue sarcomas [5]. Common anterior mediastinal tumors are known as the "4 Ts": thymoma, teratoma, thyroid tumors, and "terrible" lymphomas, which are imaged using CT or MRI, then confirmed with a biopsy and immunohistochemical staining (IHS).

MPNST histology typically demonstrates a high mitotic index, atypical spindle cells with nuclear pleomorphism, and areas of palisading necrosis [3]. IHS for MPNST would stain positive for vimentin (indicating mesenchymal origin), with possible S-100 positivity, indicating the malignant transformation of schwannoma [1,3]. In particular, loss of H3K27me3 expression has been implicated as a diagnostic marker for MPNST and as an indicator of poor prognosis and survival [1,2].

There is minimal literature documenting this condition. To the best of our knowledge, only four cases of anterior mediastinal MPNST in patients without NF1 have been reported in English literature. In Kalra et al., an 18 x 16 x 10 cm tumor was surgically removed from a 46-year-old male with no adjuvant treatment [5]. In Koezuka et al., a 10 x 10 x 8.0 cm tumor originating from the phrenic nerve was surgically removed from a 28-year-old [6]. Shimoyama et al. reported a 17 x 12 x 9.0 cm tumor surgically resected in a 75-year-old male; however, the patient died of lung metastases several years later [4]. Rais et al. used a multimodal approach to treat a 77-year-old woman's 11-cm tumor with Adriamycin to reduce tumor size by 30%, then surgically excised and treated with external beam radiation three years later, with no recurrence [7]. IHS markers are summarized in Table 1. The patient in this case had the largest tumor in the literature, measuring 9.0 x 20.5 x 9.2 cm, and it shrank successfully with chemotherapy.

Neoadjuvant chemotherapy is defined as the use of chemotherapy before surgery [8]. In this patient's case, the initial tumor was extremely large at 20.5 cm and was a poor candidate for surgical resection due to its mass effect on adjacent cardiopulmonary structures, especially the ascending aorta and pulmonary vessels. Removal of the tumor has the potential to cause serious vascular implications, such as cardiogenic shock and death. Thus, a chemotherapy regimen was attempted and shown to be successful in shrinking the tumor to alleviate clinical symptoms and potentially allow this patient to become a candidate for surgical resection. Doxorubicin and ifosfamide, the chosen chemotherapy regimen for this tumor, are standard drugs used in the treatment of soft tissue sarcomas [8,9]. Suggested dosages for doxorubicin start at 75 mg/m2 [9]. However, alternative options for chemotherapy include the use of epirubicin, dacarbazine, gemcitabine, docetaxel, and cyclophosphamide, among others [8-10].

## Conclusions

In summary, we describe a case of sporadic MPNST in the anterior mediastinum that responded to neoadjuvant doxorubicin, ifosfamide, and mesna-based chemotherapy. Although rare, MPNST tumors should be considered during the differential diagnosis of anterior mediastinal masses in addition to the "terrible Ts." Neoadjuvant chemotherapy was shown to have significantly shrunk the tumor from 20.5 cm to 9.0 cm, showing a good response compared to the literature, which largely used surgery to treat the patient. In addition, the patient, in this case, showed improved symptoms and quality of life.

## References

[1] Grobmyer SR, Reith JD, Shahlaee A, Bush CH, Hochwald SN (2008). Malignant peripheral nerve sheath tumor: molecular pathogenesis and current management considerations. J Surg Oncol.

[2] Cleven AH, Al Sannaa GA, Briaire-de Bruijn I (2016). Loss of H3K27 tri-methylation is a diagnostic marker for malignant peripheral nerve sheath tumors and an indicator for an inferior survival. Mod Pathol.

[3] Ralli M, Singh S, Hasija S, Verma R (2015). Intrathoracic malignant peripheral nerve sheath tumor: histopathological and immunohistochemical features. Iran J Pathol.

[4] Shimoyama T, Yoshiya K, Yamato Y, Koike T, Honma K (2009). Long-term survival after removal of a malignant peripheral nerve sheath tumor originating in the anterior mediastinum. Gen Thorac Cardiovasc Surg.

[5] Kalra B, Kingsley PA, Bedi HS, Kwatra KS, Negi P (2014). Malignant peripheral nerve sheath tumor of the anterior mediastinum: a rare presentation. Rare Tumors.

[6] Koezuka S, Hata Y, Sato F, Otsuka H, Makino T, Tochigi N, Iyoda A (2014). Malignant peripheral nerve sheath tumor in the anterior mediastinum: A case report. Mol Clin Oncol.

[7] Rais G, Maidi M, Rais F (2020). Successful management of intrathoracic phrenic malignant peripheral nerve sheath tumor by multimodal treatment. J Med Cases.

[8] Mangla A, Yadav U (2022 ). Leiomyosarcoma. https://www.ncbi.nlm.nih.gov/books/NBK551667/.

[9] Ratan R, Patel SR (2016). Chemotherapy for soft tissue sarcoma. Cancer.

[10] Walczak BE, Irwin RB (2013). Sarcoma chemotherapy. J Am Acad Orthop Surg.

